# Supplementary material for: Wastewater Genomic Surveillance Captures Early Detection of Omicron in Utah
Source: Microbiol Spectr. 2023 May 8;11(3):e00391-23. doi: 10.1128/spectrum.00391-23 (PMC10269515; doi:10.1128/spectrum.00391-23)
Supplement: Supplemental file 4 — Supplemental material. Download spectrum.00391-23-s0004.docx, DOCX file, 7.6 MB [file spectrum.00391-23-s0004.docx]

**Supplemental Methods:**

Flow-Population Normalization

qPCR results were normalized by both the average wastewater flow during the sampling period and the estimated population of the sewershed as follows:

$$((concentration (copies/mL) * 1,000 * flow (liters/day)) \div population) \div1,000,000$$

This yields units of millions of gene copies per person per day. The final division by 1,000,000 is simply to move the results into a more intuitive and easily interpreted range. While not a universal procedure, such normalization is common among wastewater surveillance programs, including at CDC.

**Supplementary Figures:**


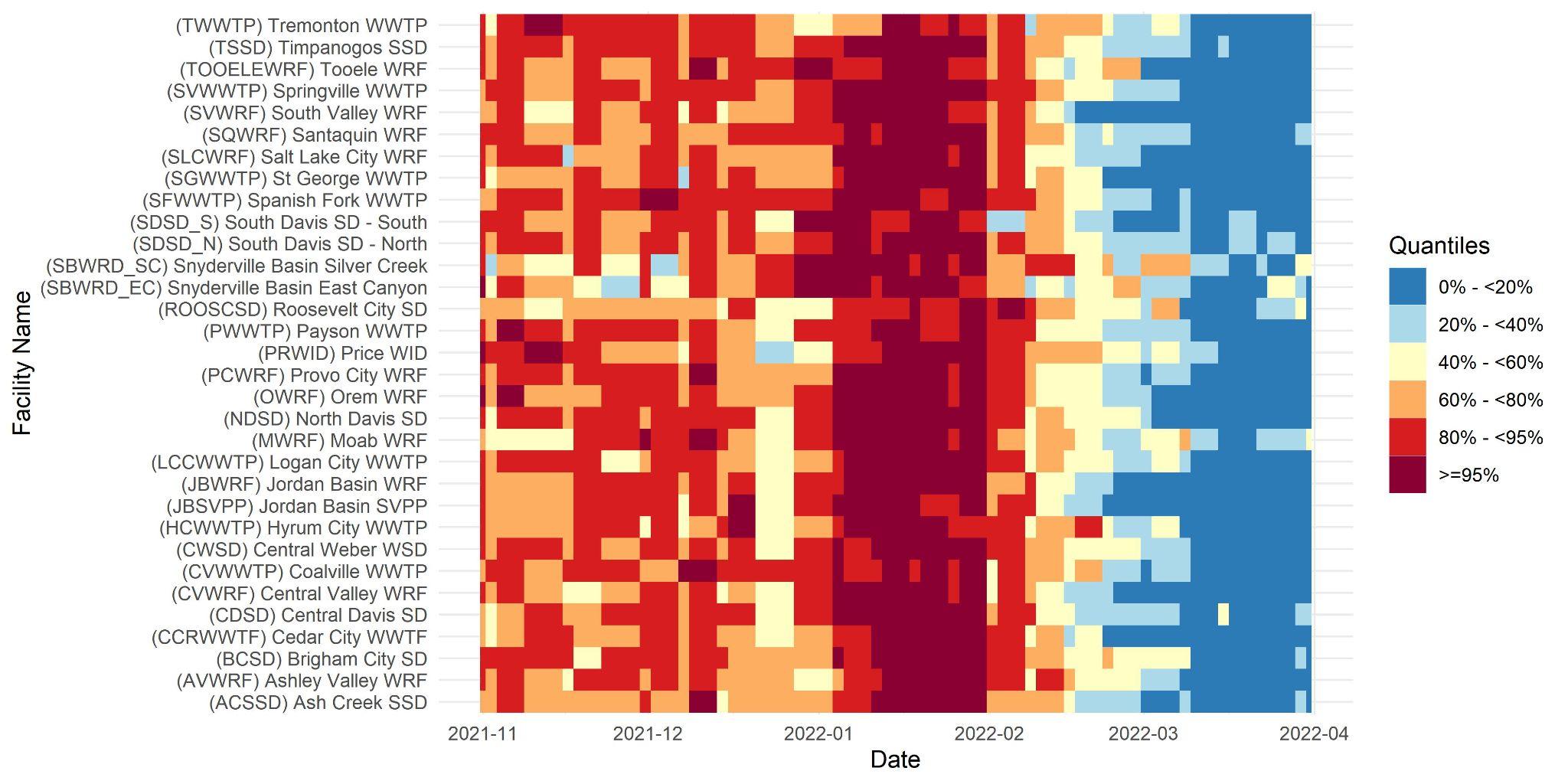


Supplementary Fig. S1. Heatmap of wastewater concentration quantiles. SARS-CoV-2 concentrations in wastewater (normalized by flow and population) were categorized into quantile bins. Bin thresholds are standard quintiles with the addition of a 95%+ bin to better emphasize the highest concentrations. The numeric thresholds were calculated on a site-specific basis on all data from July 1, 2020 to September 22, 2022, although only data relevant to the current manuscript is displayed.


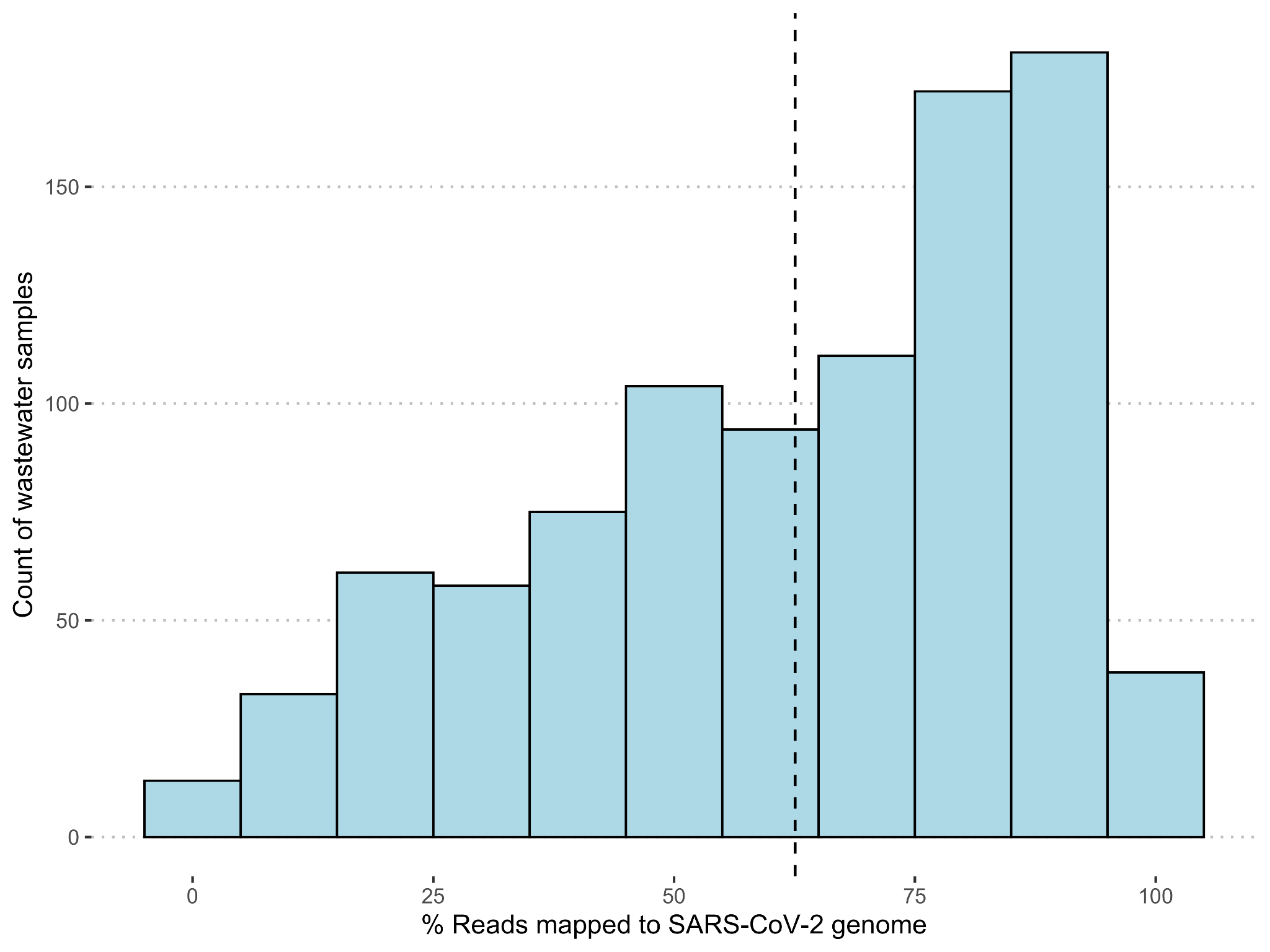


Supplementary Fig. S2. Histogram of the number of wastewater samples and their percent reads mapped to the SARS-CoV-2 genome. The black dashed line indicates the mean percentage of mapped reads.


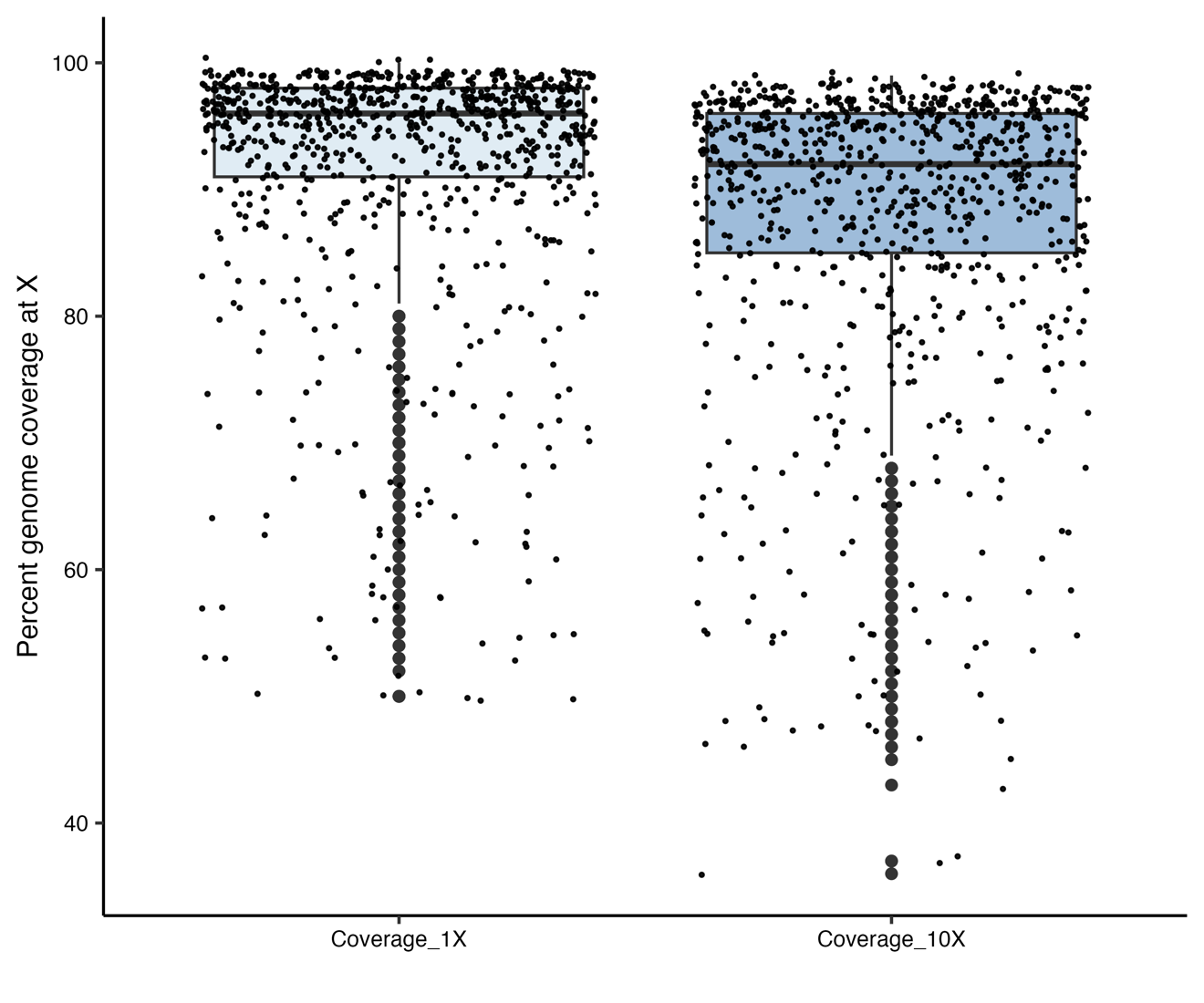


Supplementary Fig. S3. Percent genome coverage at 1X and 10X sequencing depth for all wastewater samples. Boxes represent the interquartile range, with solid lines as medians.


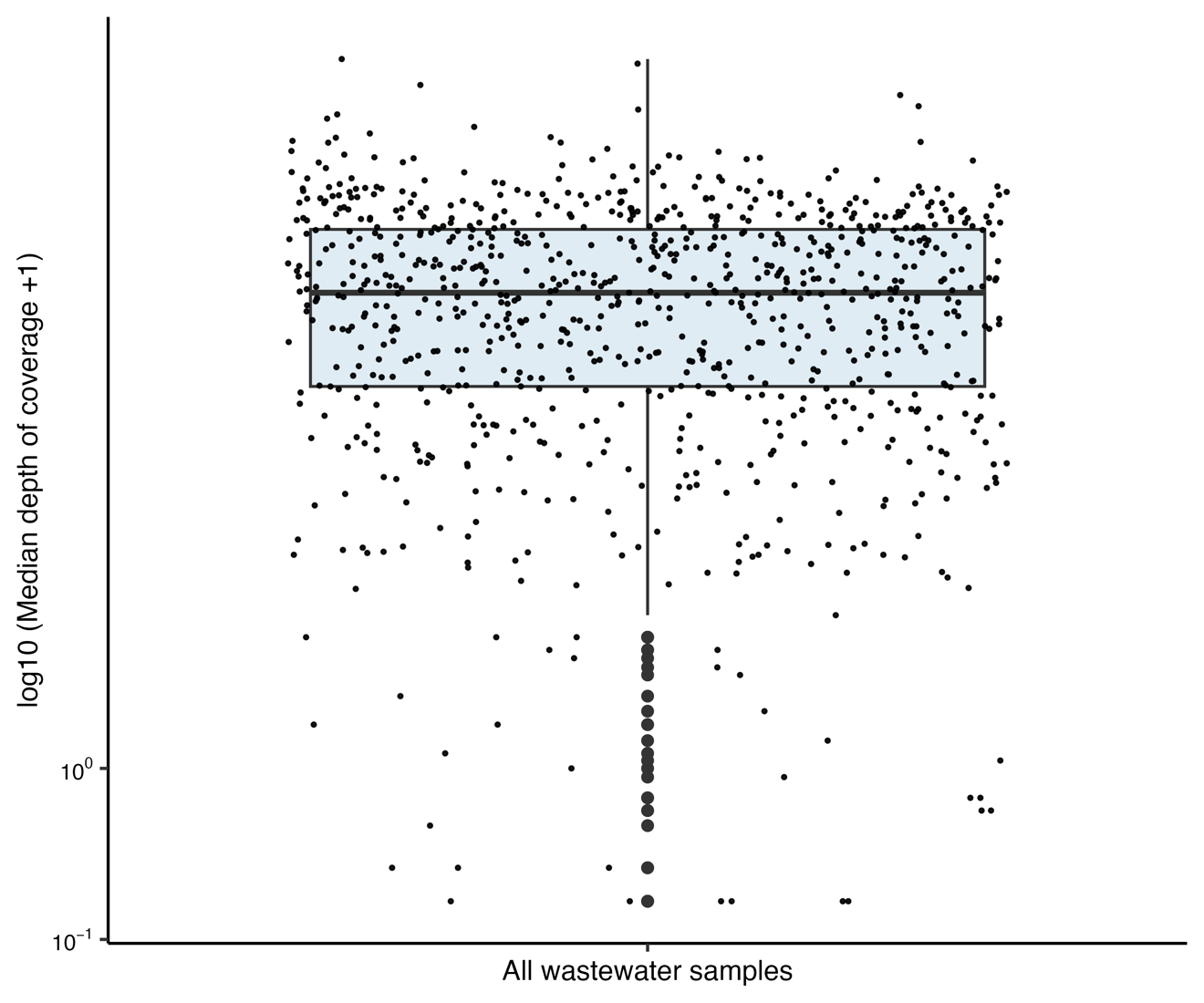


Supplementary Fig. S4. Median depth of coverage for all wastewater samples. Boxes represent the interquartile range, with solid lines as medians.


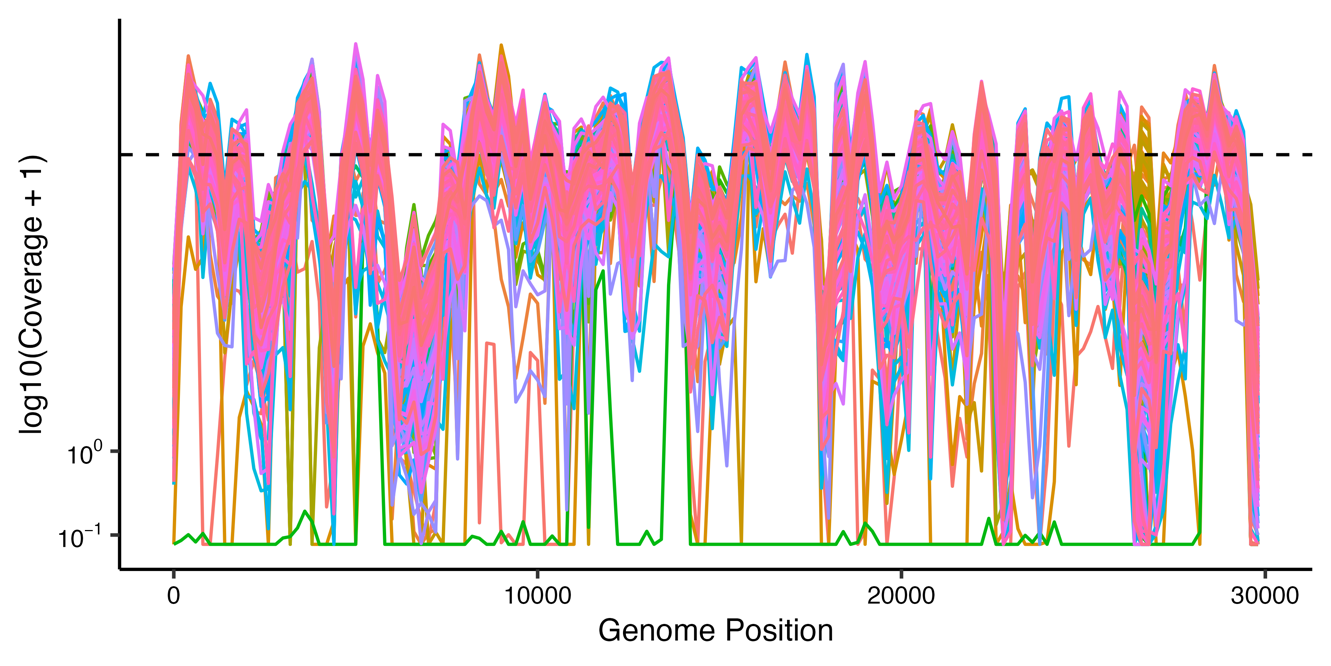


Supplementary Fig. S5. Example showing the depth of coverage across the SARS-Cov-2 genome for 184 wastewater samples collected in January 2022 from a single sequencing run and are generally representative of data from the time period included in the study.


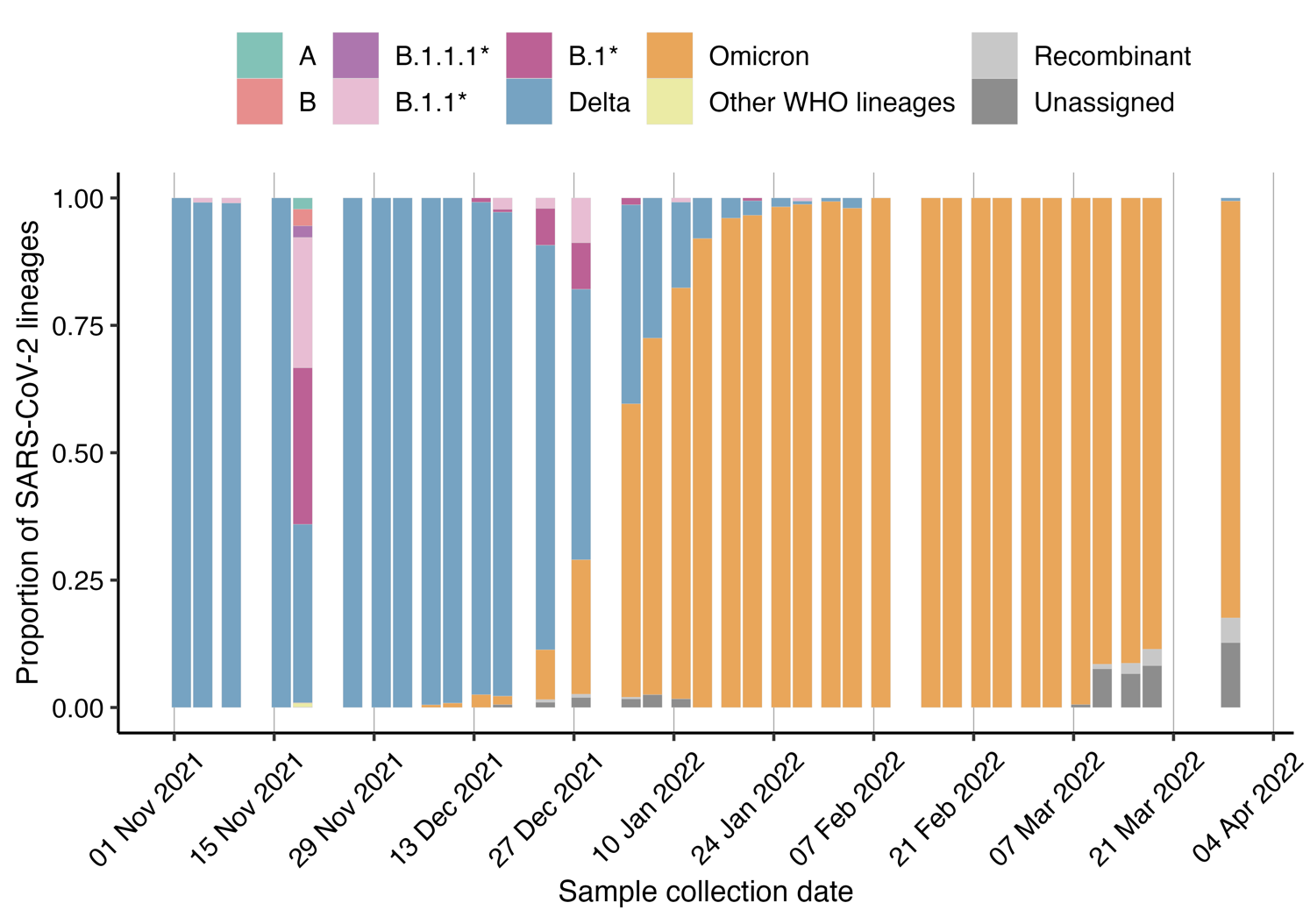


Supplementary Fig. S6. Proportion of all SARS-CoV-2 lineages in wastewater samples from November 2021 to March 2022. Detailed lineage breakdown is shown in this figure compared to Fig 2. in the main text.


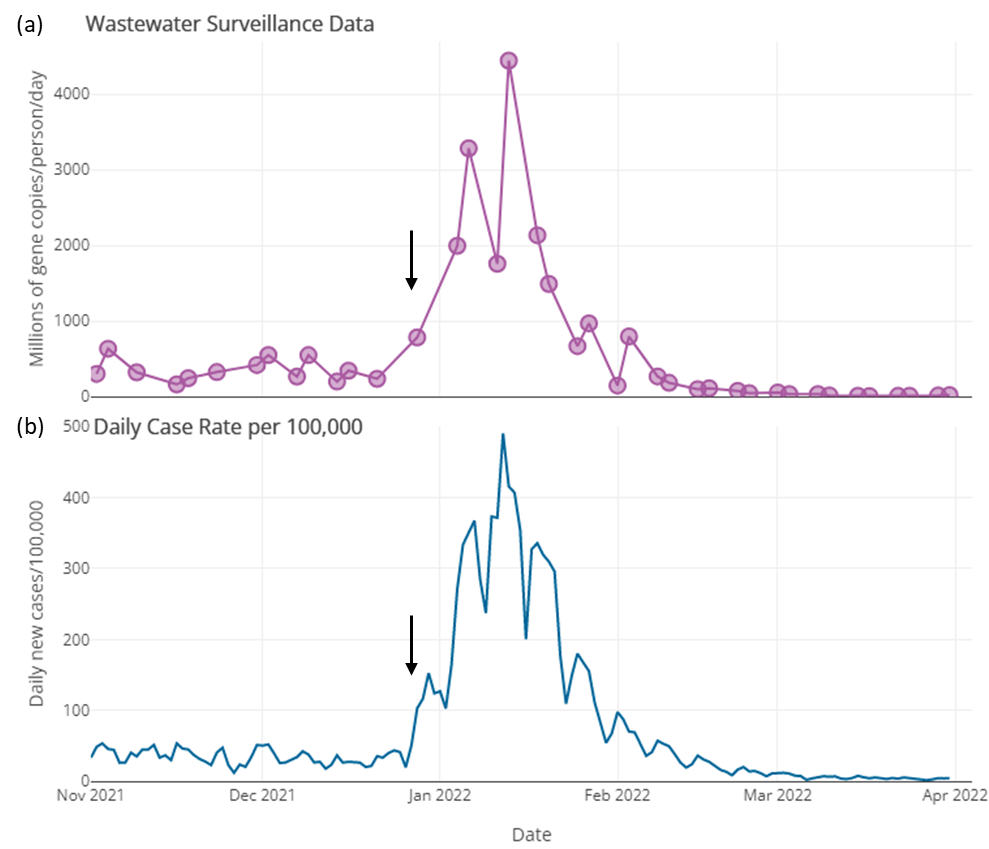


Supplementary Fig. S7. Example wastewater surveillance data and sewershed-associated case rates. The displayed data are from the (CVWRF) Central Valley WRF partner facility, and are generally representative of data from the time period included in the study. Vertical black arrows indicate the approximate start of the Omicron wave in late December 2021.

(a) Wastewater surveillance data. Data generated by UPHL in gene copies/mL of raw wastewater was normalized by estimated population and average flow during the sampling period (details in Supplementary methods). (b) Daily sewershed associated case rates per 100,000. All COVID-19 cases in Utah are geocoded by sewershed.


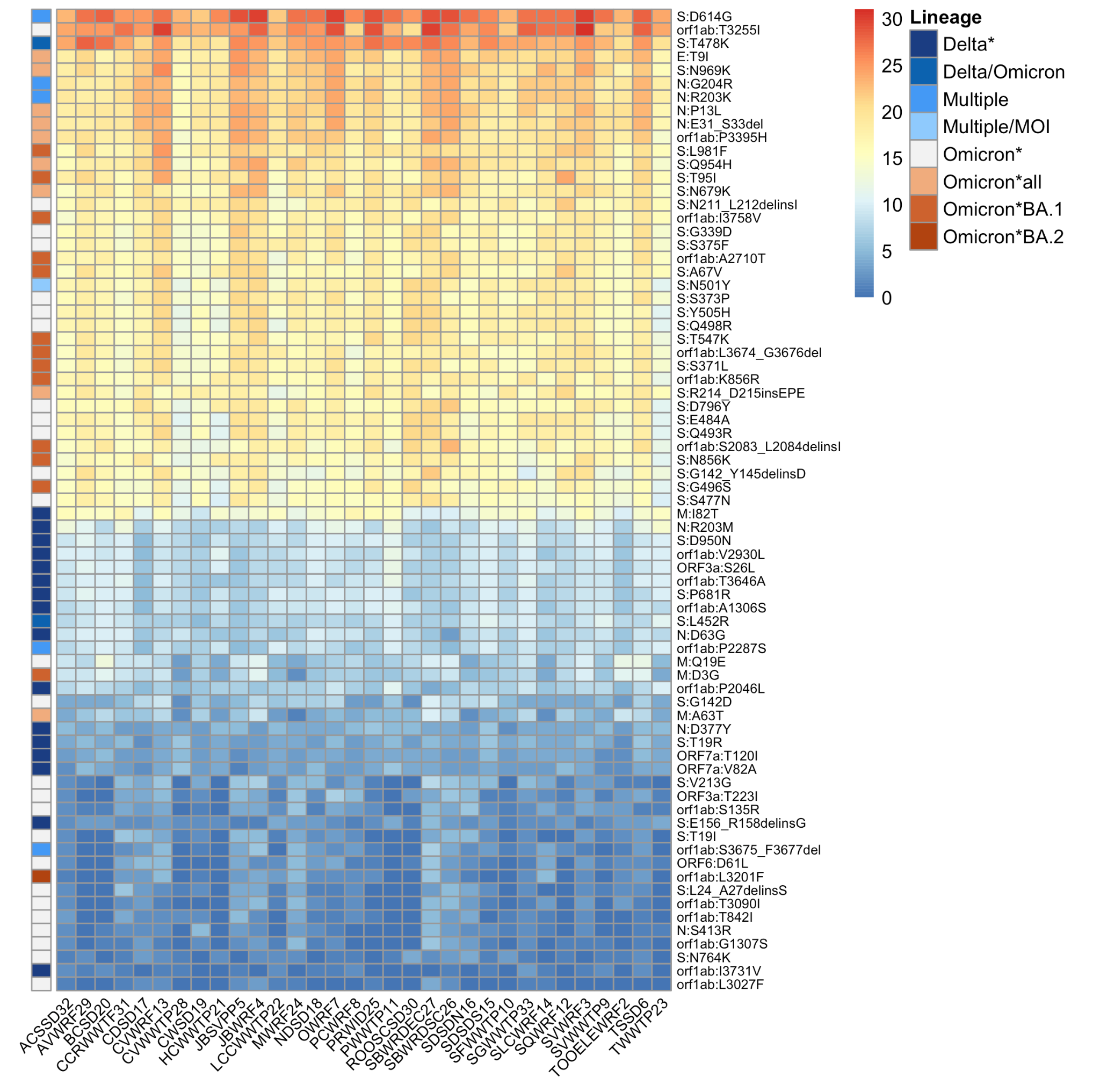


Supplementary Fig. S8. Heatmap of the frequency of all amino acid changes during November 2021 to March 2022 for each sewershed. Colors in the matrix range from cool blues representing low frequency to warm orange representing high frequency. The amino acid changes specific to a SARS-CoV-2 lineage are color coded on the left bar of the heatmap – ‘Multiple’ refers to mutations shared between multiple SARS-CoV-2 lineages and ‘Multiple/MOI’ refers to ‘mutations of interest’ shared between multiple SARS-CoV-2 lineages. Data shown here has been truncated to amino acid changes that were detected 10 or more times during the study period.


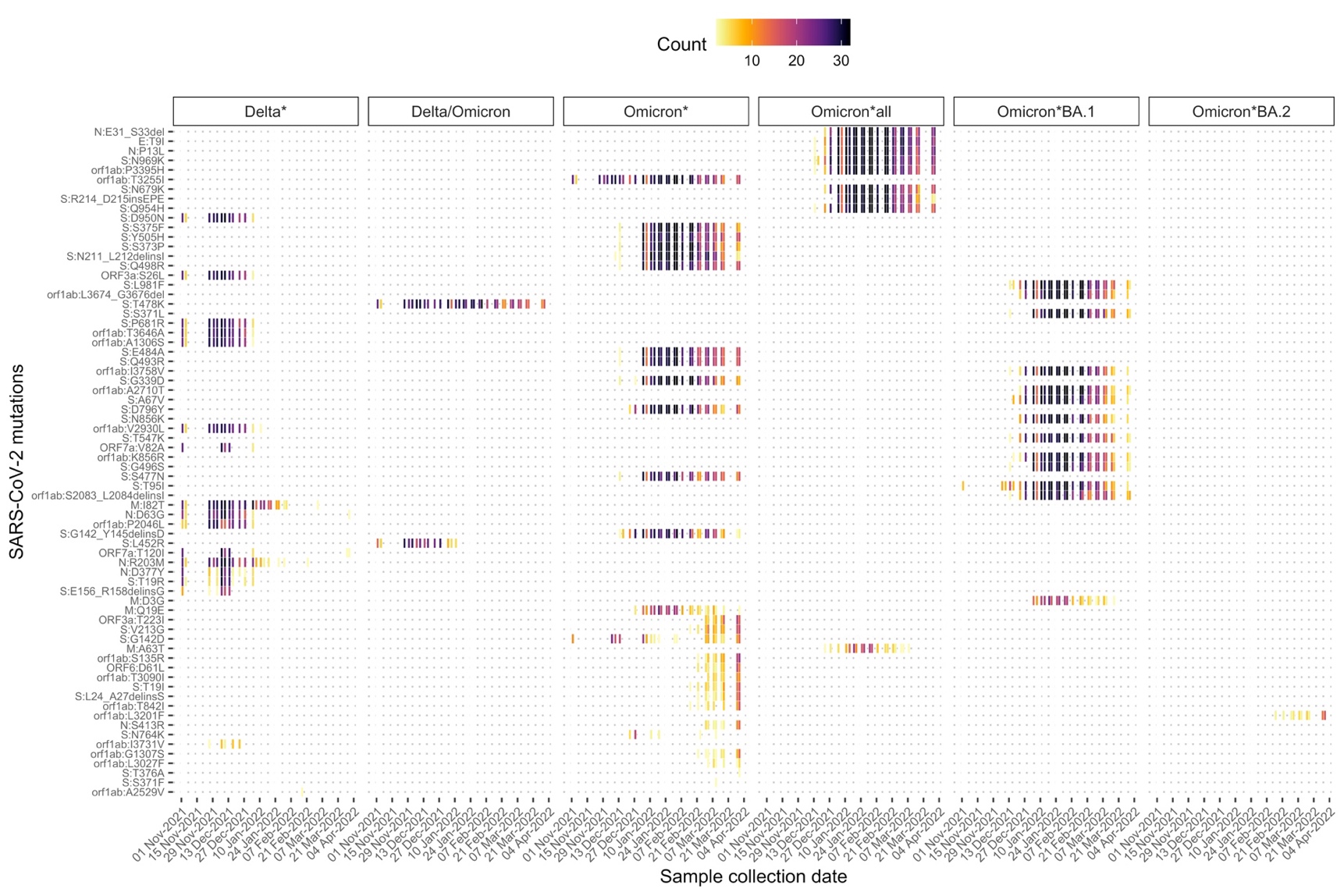


Supplementary Fig. S9. Heatmap showing the temporal changes in the frequency of SARS-CoV-2 amino acid changes from November 2021 to March 2022. Amino acid changes that were detected only once during the study period are not shown. Colors in the matrix range from light yellow/reds representing low frequency to dark blues/violet representing high frequency. The asterisk in the lineages represents mutations exclusive to that lineage. For example, Omicron*all is used to represent mutations that are found in all Omicron lineages and it sub-lineages.
